# High magnetic field induced otolith fusion in the zebrafish larvae

**DOI:** 10.1038/srep24151

**Published:** 2016-04-11

**Authors:** Patricia Pais-Roldán, Ajeet Pratap Singh, Hildegard Schulz, Xin Yu

**Affiliations:** 1 High Field Magnetic Resonance Department, Max Planck Institute for Biological Cybernetics, Tübingen, 72076, Germany; 2 IMPRS for Cognitive and Systems Neuroscience, Tübingen, 72076, Germany; 3 Max Planck Institute for Developmental Biology, Tübingen, 72076, Germany

## Abstract

Magnetoreception in animals illustrates the interaction of biological systems with the geomagnetic field (geoMF). However, there are few studies that identified the impact of high magnetic field (MF) exposure from Magnetic Resonance Imaging (MRI) scanners (>100,000 times of geoMF) on specific biological targets. Here, we investigated the effects of a 14 Tesla MRI scanner on zebrafish larvae. All zebrafish larvae aligned parallel to the B_0_ field, i.e. the static MF, in the MRI scanner. The two otoliths (ear stones) in the otic vesicles of zebrafish larvae older than 24 hours post fertilization (hpf) fused together after the high MF exposure as short as 2 hours, yielding a single-otolith phenotype with aberrant swimming behavior. The otolith fusion was blocked in zebrafish larvae under anesthesia or embedded in agarose. Hair cells may play an important role on the MF-induced otolith fusion. This work provided direct evidence to show that high MF interacts with the otic vesicle of zebrafish larvae and causes otolith fusion in an “all-or-none” manner. The MF-induced otolith fusion may facilitate the searching for MF sensors using genetically amenable vertebrate animal models, such as zebrafish.

There are profound clinical applications of magnetic resonance imaging (MRI), where genetic and chemical tools have helped researchers develop endogenous proteins and exogenous contrast molecules that can be specifically detected using MRI^1^,^2^,^3^,^4^,^5^,^6^,^7^. Besides the extensive efforts to create exogenous biological markers for MRI, much work has also been done to investigate endogenous magnetic field (MF) receptors that could potentially interact with biological processes when exposed to a high MF inherent to MRI scanners. MRI scanners for both clinical practice (1.5 T–7 T) and basic research (7 T to 21 T) have a MF that ranges from 20,000 to more than 200,000 times higher than the geomagnetic field (GeoMF, ~0.3 Gs–0.6 Gs). Thus, it provides a unique platform to uncover interactions of the high MF with certain biological processes in living organisms.

The existence of biological MF sensors is supported by previous magnetoreception studies^8^. Extensive work has shown how different species detect and respond to the geoMF^9^,^10^,^11^,^12^. However, it remains challenging to study the underlying biological mechanism of geo-magnetoreception^13^. Three biophysical mechanisms that potentially underlie interactions between biological processes and the MF have been proposed: 1. radical pair interactions^12^; 2. magnetite associated processes^10^; and 3. magneto-electro-reception^14^. Although extensive work has been done in non-human animals, few reports have shown direct interactions between the MF and the human body^15^. In the high MF domain, vertigo and nystagmus have been reported in humans undergoing MRI, indicating that there might be a certain susceptibility of the human vestibular system to the MF^16^,^17^,^18^. Interestingly, the inner ear lagena, which projects to vestibular nuclei, has been proposed as a target to mediate MF perception in the avian brain^19^. These reports led us to consider the vestibular system as a target for high MF interactions. Previous studies on the swimming patterns of zebrafish have demonstrated that this vertebrate bears magnetoreception capabilities^20^,^21^. In this study, we characterized the impact of MF exposure on the vestibular system of zebrafish larvae in an MRI scanner.

The zebrafish is a genetically tractable model vertebrate, widely used on account of its external fertilization and its rapid and well-studied development^22^,^23^,^24^. The inner ear of zebrafish larvae consists mainly of three semicircular canals and two ear stones called otoliths which are attached to two maculae^25^ (Fig. 1). Otoliths are crystalline structures of calcium carbonate (CaCO_3_) that transmit acceleration forces and sound vibrations to the ciliary bundles of macular hair cells, thereby contributing to the vestibular function of the animal. Zebrafish inner ear provides an elegant and well-studied platform to search for MF-induced modifications. The present study demonstrated an unexpected fusion of otoliths in the zebrafish larvae after MF exposure in a 14T MRI scanner.

## Results

### High MF-exposure leads to a fusion of otoliths in Zebrafish larvae

**Figure 1:**
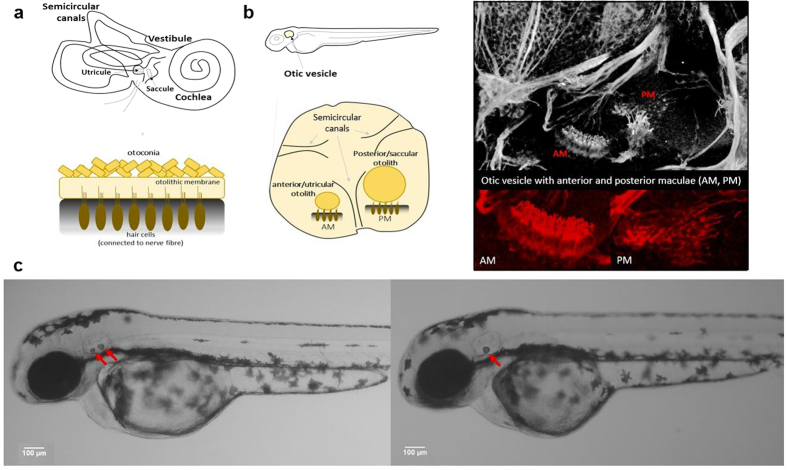
Otolith fusion in zebrafish larvae exposed to high MF. (**a**) Scheme of the human inner ear. (**b**) Scheme of the zebrafish larva inner ear (left) and microscopy image of the otic vesicle stained with anti-acetylated tubulin antibody (right). AM: anterior macula hair cells, PM: posterior macula hair cells. The otoliths (dark yellow circles) are tethered to the wall of the otic vesicle by hair cells (in brown). The otoliths equivalent structures in the human inner ear are multiple crystals called otoconia, which do not contact the hair cells directly but are embedded in an otolithic membrane. (**c**) Representative phenotype of control (left, two otoliths, red arrows) and MF-exposed (right, single otolith, red arrow) larvae.

**Figure 2:**
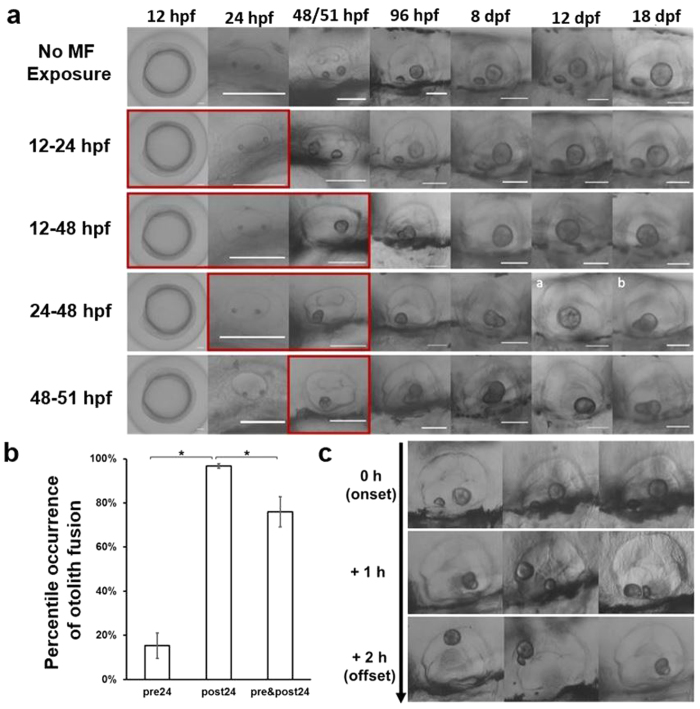
Time dependency of the MF response. (**a**) Representative phenotypes of the otic vesicle of larvae exposed to a 14 T MF for different lengths of time (Y axis), at different developmental points (X axis) up to 18 days post fertilization (dpf). Pictures in the horizontal series taken from siblings exposed during the same specific time window (not necessarily the same embryo). Most of the larvae exposed to the MF before reaching the age of 24 hours post fertilization (hpf) were able to form two otoliths and kept them until the end of our study. Exposures after 24 hpf resulted in the fusion of both otoliths, which persisted until, at least, 18 dpf. The first column shows an embryo at 12 hpf (otic vesicle can still not be identified). Red boxes indicate the duration of MF exposure. Scale bar is 100 μm. (**b**) Prevalence of the otoliths-fused phenotype in embryo/larvae exposed before 24 hpf (“pre 24”, 15.3 ± 5.8%, n = 327, number of trials = 7), after 24 hpf (“post 24”, 96.9 ± 0.9%, n = 1130, number of trials = 27) or exposed from before 24 hpf to after 36 hpf (“pre&post 24”, 76.6 ± 7.4%, n = 649, number of trials = 18). (*) For “pre 24(hpf)” vs. “post 24(hpf)”, p < 0.001; for “post 24(hpf)” vs. “pre&post 24(hpf)”, p < 0.01. (**c**) Otolith fusion from three randomly chosen zebrafish larvae (3 dpf) at 3 different time points of MF exposure (before exposure (2 otoliths), 1 hour after onset of MF-exposure, and 2 hours after onset of MF-exposure).

To characterize the effect of the MF on the development of the otolith organ, we first exposed zebrafish embryos (aged 10 hours post fertilization, hpf) to a 14T MF, at a temperature of 28–29 °C for 62 hours (the embryos were 72 hpf when the exposure ended). In contrast to control embryos, which exhibited two otoliths in each ear at the geoMF (0.3–0.6 Gs^11^), only one otolith was detected in larvae that were exposed to a high MF (Fig. 1c). Otoliths are usually formed between 16 hpf and 24 hpf 26. A single otolith phenotype was consistently observed in larvae that were exposed to a high MF after the formation of the two otoliths, as depicted in rows 3 to 5 of Fig. 2a,b (89.5 ± 3.4% of animals exposed during a period of time that covered the post(otolith)-formation stage, n = 1779). These larvae maintained a single otolith throughout the course of our study (up to 18 days post fertilization (dpf)) unlike the control group, which always displayed two otoliths (Fig. 2a, 1^st^ row). However, in larvae exposed to a high MF between 8 and 24 hpf (before otolith formation), the occurrence of a single otolith phenotype was significantly reduced (15.3 ± 5.8%, n = 327) (for pre 24 hpf vs. post 24 hpf, p < 0.001; Fig. 2b). Most larvae that were exposed until 24 hpf (the early exposure group, 2^nd^ row of Fig. 2a) developed two otoliths (84.7 ± 5.8%), like the controls (Fig. 2a, 1^st^ row). This result suggests that a single otolith phenotype was induced during a high MF exposure and not during the post-exposure period. In addition, this suggests that the single otolith phenotype might be the result of a fusion event between the two otoliths. To test this possibility, we exposed the larvae to a high MF and imaged their otoliths at regular intervals (every hour). The two otoliths detached from the walls of the otic vesicle within 1 hour of exposure to a high MF, and their fusion could be readily observed 2 hours after the onset of high MF exposure (Fig. 2c). Thus, these results show a robust high MF-induced phenotype of fused otoliths when 24 hpf larvae are exposed for at least 2 hours.

### The probability of otolith fusion in Zebrafish larvae exposed to the MF increases with the intensity of the MF

**Figure 3:**
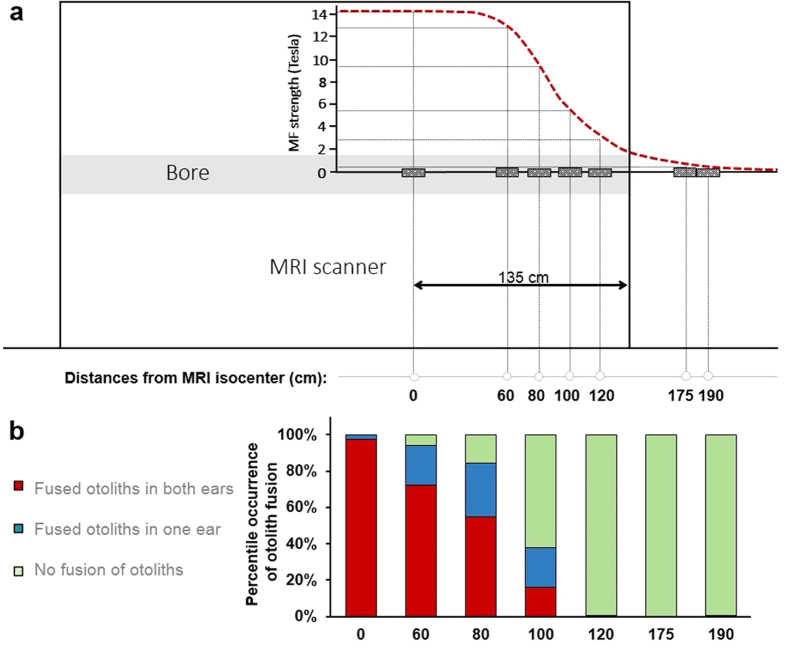
MF strength dependency. (**a**) Scheme of the MF dependency experiment. The red dash line indicates the approximate MF strength along the bore of the magnet. Pattern-filled boxes represent the location of petri dishes containing zebrafish larvae. (**b**) The graph shows the percentage of 3 dpf larvae (2h of MF exposure) exhibiting otolith fusion (Y axes) at different distances from the iso-center. For this experiment, n = 124 (6 trials); 201 (11 trials); 274 (12 trials); 290 (12 trials); 323 (12 trials); 154 (6 trials); 198 (6 trials) for the distances 0 cm; 60 cm; 80 cm; 100 cm; 120 cm; 175 cm; 190 cm respectively. For the positions 0, 60, 80, 100, 120, 180 and 190 cm from the iso-center, the percentage of larvae with bilateral fusion (both ears showing the fusion phenotype) was 97.6 ± 1.0%, 72.6% ± 8.9%, 54.7 ± 7.2%, 15.9 ± 5.0%, 0.0 ± 0.0%, 0.0 ± 0.0%, and 0.5 ± 0.5%, respectively; the percentage of larvae with unilateral fusion (only one of the two ears showing the fusion) was 2.4 ± 1.0%, 21.4 ± 6.3%, 29.6 ± 4.7%, 22.1 ± 4.4%, 0.6 ± 0.3%, 0.0 ± 0.0%, and 0.0 ± 0.0%, respectively.

The MF strength rapidly decreases from the edge of the magnet core of MR scanners (Fig. 3a). To examine the MF effect on the fusion of otoliths at different strengths, we placed the dishes containing zebrafish larvae at different distances from the iso-center of the 14 T MR bore. The prevalence of the otolith fusion decreased as a function of the distance from the iso-center of the 14 T scanner (Fig. 3b). No fusion of otoliths was reliably observed for larvae exposed to 3 T MF. The fusion of otoliths occurred in less than 40% of the larvae when exposed to a MF under 6 T (less than 16% were affected in both ears), and the incidence of otolith fusion rose with increase in MF intensity from 9 T (84% affected - 55% bilaterally-) to 14 T (100% affected - 98% bilaterally). The MF dependency of the otolith fusion process further verified the susceptibility of the otolith organ under a high MF.

### Zebrafish larvae with fused otoliths after the MF exposure exhibit an aberrant swimming behavior

**Figure 4:**
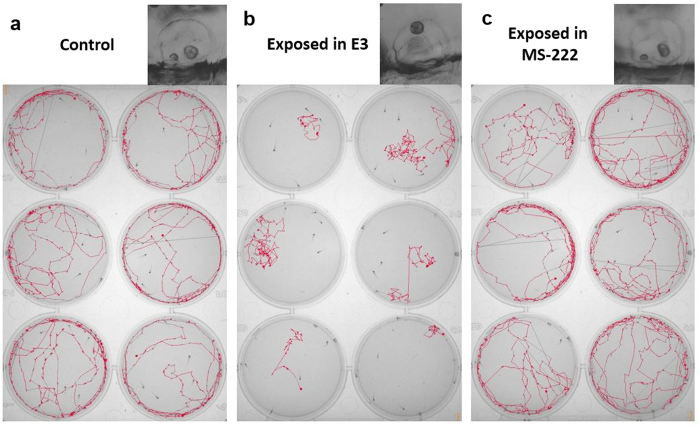
Swimming behavior of zebrafish larvae. (**a**) Six different swimming tracks of control larvae. (**b**) Swimming tracks of six different larvae with MF-induced otolith fusion. (**c**) Swimming track of six larvae with two otoliths that were previously exposed to the MF with the presence of MS-222 (anesthetized). Inset is the representative phenotype of the otolith fusion from each group. All figures represent 3 dpf larvae. Supplementary videos 1 and 2 showed the real time track videos of control larvae and larvae exposed in normal embryonic medium (E3) to the MF.

The resulting otolith fusion observed under microscope in the larvae that were exposed to the MF was accompanied with a subsequent abnormal swimming behavior, consisting mainly of a reduction in swimming activity, a circling motion and a failure to maintain normal posture (Supp. Videos 1 and 2). In larvae with unfused otoliths (e.g. larvae exposed to a high MF in MS-222 or agarose medium), we did not observe altered swimming patterns post-MF exposure. This result suggests that an alteration in swimming ability and/or equilibrioception is a direct consequence of the otolith fusion. Figure 4 shows the swimming movements of larvae from three different groups (control, exposed to MF in normal E3 and exposed to MF while anesthetized –swimming assessed in fresh E3 medium one day after the MF exposure-). The emergence of the MF-induced otolith fusion and the subsequent circling behavior while swimming resembles zebrafish circler mutants^27^.

The zebrafish larvae were also recorded during the MF exposure. Under the high MF, both awake and anesthetized zebrafish larvae aligned parallel to the B_0_ field of the scanner, i.e. the horizontal direction of the static MF along the magnet bore (Supp. Fig. 4). Larvae exposed under the MF in normal embryonic medium were observed to be able to swim in all directions, but they always returned to a default position oriented with the high MF (Supp. Video 3). The body of the anesthetized larvae aligned parallel to the MF and this orientation remained during the whole exposure time (Supp. Video 4). Previous studies performed in adult zebrafish exposed to a strong MF (11.7 T) also showed a change in the swimming behavior, though passive alignment of the bodies in a specific direction with respect to the MF was not reported^21^. Besides, these adult fish only showed a disruption in the swimming patterns inside the bore of the magnet and not after the exposure. It remains to be elucidated if otolith fusion occurs in adult zebrafish under high MF.

### Conditions of exposure can predetermine the occurrence of MF-induced otolith fusion in Zebrafish larvae

**Figure 5:**
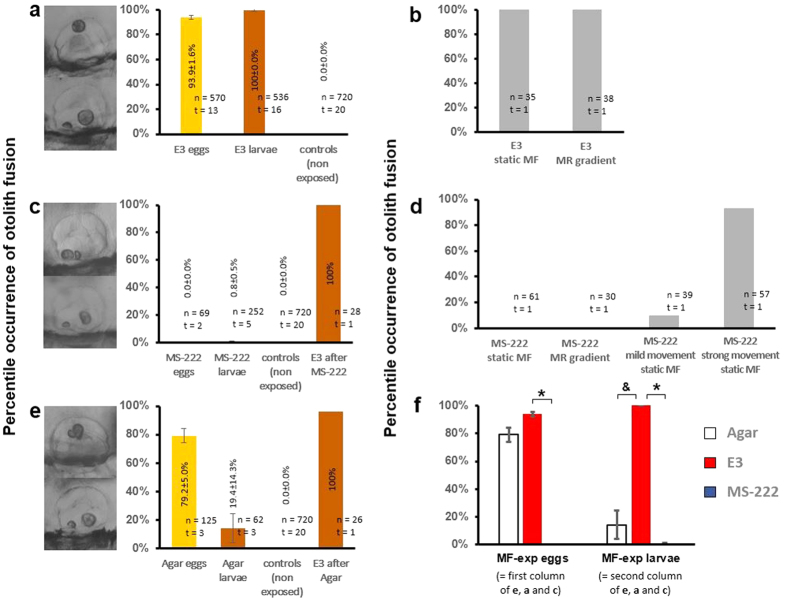
MF-induced otolith fusion under different environmental conditions. (**a,c,e**) show the response from larvae that were exposed in dishes containing either embryonic medium (E3) (**a**), anesthetic (Tricaine MS-222) (**c**), or a more solid medium (0.8% agarose) (**e**). For these experiments, larvae with chorion (labeled “egg”, yellow-colored) or hatched larvae (labeled “larvae”, orange-colored), were exposed under MF for at least 2 hours. Controls included eggs and larvae embedded in the same medium and maintained in a fish incubator. (**b**) shows the response of larvae exposed to the 14 T MF with and without additional MF gradients generated with an MR sequence. (**d**) shows the response of anesthetized larvae that were either exposed to the normal 14T MF, the MF with additional gradients or exposed to the normal 14T MF while passively moved by a mild and a stronger external airflow. (**f**) summarizes the statistical analysis of otolith fusion at different conditions (*means p < 0.001; ^&^means < 0.05). Insets besides the Y axes showed the representative larvae with fused otoliths. In all graphs, n = number of samples and t = number of trials carried out.

The incidence of otolith fusion observed after MF exposure decreased in larvae that were exposed restrained in agarose (0.8% low-melting agarose in E3) or anesthetized with tricaine mesylate (0.04% MS-222 dissolved in the E3 medium). As aforementioned, MF exposures in a normal embryonic medium led to a phenotype characterized by fused otoliths in larvae after 24 hpf (100% ± 0.0% showing the otolith fusion in at least one ear, n = 560, Fig. 5a). However, the percentage of otolith fusion was significantly reduced in larvae that were exposed to a high MF while embedded in agarose (19.4 ± 14.3%, Fig. 5e) or kept in E3 medium with dissolved anesthetic (0.8 ± 0.5%, Fig. 5c) (Fig. 5f shows, on the right, the significant difference between larvae exposed in agar, E3 and MS-222). For those zebrafish larvae either anesthetized or embedded in agarose that did not undergo fusion of otoliths under MF, the anesthetic was washed out and the agarose was replaced by normal E3 medium to repeat the MF exposure in standard conditions. Re-exposure of these larvae in E3 medium to the MF resulted in otoliths fusion (fourth column of Fig. 5c,e). This result suggested that anesthetics or embedding of the larvae in agarose involve an *in situ* preventive effect of the otolith fusion under MF.

One plausible explanation for the prevention of the MF-induced otolith fusion in anesthetized or agarose-embedded larvae is a lack of motion, which might prevent a certain interaction between the otoliths (or their tether to the otic vesicle) and the MF. Two experiments were performed to verify the underlying factors contributing to otolith fusion. First, we exposed anesthetized larvae to the MF with a passive movement triggered by a constant airflow to the medium. A mild airflow moved the larvae around the dish without changing their alignment with the MF (translation occurred, but rotation of the body was not observed. Supp. Video 5). Only 4 of 39 anesthetized larvae showed otolith fusion after 2 hours of exposure (Fig. 5d, third column). In contrast to this translational movement, a stronger airflow, provided to a second petri dish with anesthetized larvae, elicited rotation of larvae bodies, disrupting the constant alignment with the static MF (Supp. Video 6). After 2 hours of exposure, 53 of 57 larvae had fused the otoliths. These results suggest that a disruption of the alignment of larvae with the MF may force the detachment of the otolith from its tether. The otolith particles in the otic vesicle may behave as a dipole and align parallel to the B_0_ field. When the orientation of the larvae mismatches the direction of the external MF (e.g. while swimming), the otolith particles may be subject to a torque. This torque might have an influence on the tether points between the otoliths and the otic vesicle, which eventually causes detachment and fusion of the otoliths. However, the high airflow could also alter the anesthetized states of the zebrafish larvae through the passive motion of the larvae bodies, as well as the oxygen contents in the medium. Rather than move the anesthetized larvae body from the main B_0_ field, we also applied a pseudo-MR sequence to constantly vary the MF gradients not only at the B_0_ longitudinal direction (z-axis), but also at the transverse plane (x and y axes), which introduced radical gradient changes in 3-dimensional space. The MR sequence implemented gradients to instantaneously alter the magnetic dipole moment that the immobilized zebrafish may experience, which could potentially mimic the torque experienced by the zebrafish swimming toward directions deviated from the B_0_ field or even by the anesthetized fish passively moved through the inhomogeneous MF. No fusion of otoliths was observed in the anesthetized larvae under this condition (Fig. 5d, second column), in contrast to the E3 medium larvae, in which the otoliths fused both under the usual 14T MF and also under the MF with additional MR gradients (Fig. 5b). Therefore, a change in the magnetic dipole moment induced by MF gradient switch during exposures alone is insufficient to induce otolith fusion. Likewise, disturbance of the larvae body alignment may not be the sufficient condition to cause otolith fusion.

A variability in the level of metabolism might also interfere with the fusion of otoliths. Anesthetized larvae, as well as those embedded in agarose, have, in addition of a restricted motion, a down-regulated or slower metabolism, partially due to reduced oxygen consumption. Indeed, embryos older than 24 hpf that were still surrounded by the chorion displayed otolith fusion under agarose but not under anesthetic conditions (Fig. 5f, left columns). The chorion provides protection for the developing fertilized egg (e.g. it offers a compartment where the embryo can move in spite of the surrounding agarose), but it also represents a physical barrier for gas exchange^28^,^29^, which might help retaining oxygen stores for the developing embryo. Also, embryonic movement (present in eggs embedded in agarose but absent in the anesthetized embryos) has been suggested to improve oxygen supplies^28^. These results indicated that both active motion and high metabolism may play crucial roles underlying otolith fusion.

### Hair cells could participate in the MF-induced otolith fusion of Zebrafish larvae

Previous reports have suggested that the activity of hair cells is critical for the formation of otoliths in zebrafish^22^,^26^,^30^. The hair cells might be directly involved in the otolith fusion. To examine whether hair cells of the zebrafish larval inner ears were altered after MF exposure, we labeled hair cells in the otic vesicles with anti-acetylated Tubulin (Fig. 1, supp. videos 7 and 8). No significant difference in the number of hair cells was detected between control animals and those exposed to high MF (anterior macula: p = 0.63, posterior macula: p = 0.17; n = 8), including the distance between anterior and posterior maculae (p value = 0.37) and their distribution in the otic vesicle (p = 0.99) (Supp. Fig. 2). These results suggested that the high MF exposure may not alter the morphology of hair cells in the inner ears of larval zebrafish.

**Figure 6:**
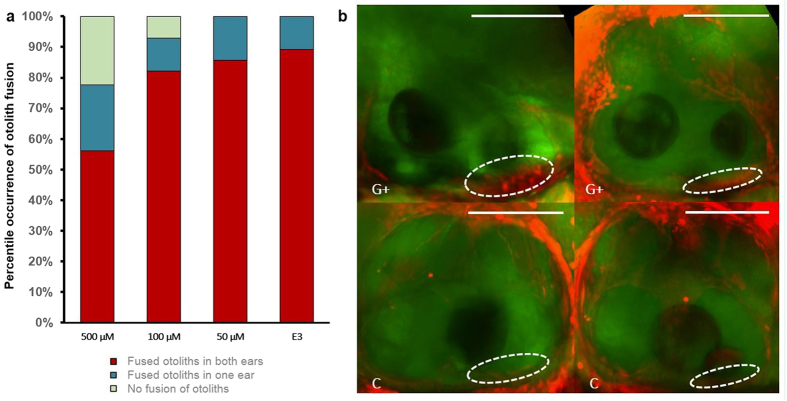
The effect of MF on otolith fusion under hair cell - compromised condition. (**a**) MF-induced otolith fusion percentage from groups of zebrafish larvae after 24 hours of treatment with different concentrations of gentamicin (500, 100, 50 and 0 μM) in E3 medium. The number of samples studied after MF exposure in this study was 139, 56, 70, and 121, treated with 500 μM, 100 μM, 50 μM and non-treated (E3 medium), respectively. (**b**) *In vivo* staining – confocal imaging of treated (G+) vs. non-treated (C) larvae. The images show the maximal intensity Z projections of bright field (green) and acridine orange (AO, red) stained larvae. Otic vesicles of 500 μM Gentamicin treated larvae show a high intensity signal coming from the apoptotic dye AO on the hair cells (presumably apoptotic cells). Dash lines represent the area corresponding to the hair cells of the anterior macula. Scale bar = 100 μm.

To examine whether hair cells contributed to the fusion of otoliths under MF, the zebrafish larvae were treated with the aminoglycoside gentamicin, which has been previously reported to induce apoptosis in zebrafish hair cells^31^,^32^,^33^,^34^,^35^. Figure 6a shows the reduced number of larvae exhibiting MF-induced otolith fusion under higher concentrations of gentamicin. Gentamicin-treated larvae that did not undergo otolith fusion after high MF exposure showed a large degree of apoptosis in hair cells of the otic vesicle when stained with the vital dye Acridine Orange. In contrast, we observed little to no Acridine Orange signal in hair cells of larvae that did not receive gentamicin treatment (Fig. 6b). Meanwhile, the larvae treated with gentamicin (100 and 500 μM) for 24 hours showed similar swimming patterns to the normal larvae when exposed under high MF (Supp. Video 9). Therefore, the lack of otolith fusion after gentamicin treatment does not seem to be caused by the lack of movement under the high MF. This result suggests that disrupted hair cells hamper, to a certain extent, the high MF-induced otolith fusion, and thus, hair cells may be directly involved in the fusion of otoliths under the MF.

## Discussion

In the present work, we observed that the fusion of two otoliths in the otic vesicle of 24 hpf zebrafish larvae occurred under high MF. After exposure under MF, the zebrafish larvae bearing single-otolith phenotype showed an aberrant swimming behavior. Meanwhile, the larvae bodies aligned parallel to the static MF in the magnetic bore. The deviation of the bodies’ magnetic orientation and normal hair cell function are shown to be directly related to the fusion of otoliths in the larvae otic vesicle. The otoliths of the affected larvae remained fused for at least 14–18 days.

The anatomical phenotype observed in high MF-exposed larvae strongly resembles the zebrafish mutant *einstein* (ein = one, Stein = stone), which is characterized by a single otolith in the otic vesicle^36^,^37^. It is worth noting that all larvae described in the present study were *wildtype*, and that they only developed single-otolith phenotype similar to *einstein* upon exposure to a high MF. Interestingly, changes in gravity have also been reported to alter inner ear morphology; for example, fish larvae kept under hyper-gravity conditions (achieved in a centrifuge) exhibited smaller otoliths^38^,^39^, and *Xenopus* larvae reared in space^40^ exhibited bigger otoliths. Previous studies have also shown a behavioral response in adult zebrafish to the geoMF^20^ and to a higher MF^21^. These studies are consistent with our observations that developing zebrafish otoliths are highly vulnerable under high MF.

The fusion of otoliths that occurs upon high MF exposure might partially result from the chemical composition of the otolith. Formation of the otic vesicle starts at 16 hpf in wild type zebrafish. At the first stage of otolith formation (18 to 24 hpf), glycogen particles (seeding particles) can be found in the otic vesicle^41^, which aggregate and form the core of the otoliths. Aggregation of these particles occurs at the kinocilia of hair cells, at both poles of the otic vesicle. After this period of time, Ca^2+^ and CO_3_^2−^ levels increase in the otic vesicle, and the initial otoliths (glycogen aggregates) become mineralized with a crystalline casing of calcium carbonate (CaCO_3_)^23^,^42^. Although the CaCO_3_ particles are diamagnetic^43^, several studies reported an increase in CaCO_3_ precipitation in water under MF (0.1 T–0.5 T)^42^,^44^. The mechanism behind the MF enhanced CaCO_3_ aggregation has not been characterized yet, but it has been suggested that electrically charged crystal nuclei could interact with the MF, with a resultant Lorentz force altering size and shape of the crystal^45^. Other proposed mechanisms include changes in electron configuration or in the shell of water molecules around ions^46^. This suggests that the magnetic susceptability of the otolith, composed mainly of calcite^43^ may allow it to sense the MF in the inner ear of zebrafish and give rise to the fusion of otoliths under high MF. Certain biological processes (possibly involving hair cells, as yet unidentified), in addition to the chemical composition of otoliths, may play a crucial role in their fusion under high MF. The magnetic character of zebrafish has been recently reported with findings of ferromagnetic aggregates in the zebrafish lateral line^47^. Although the biophysical basis of the alignment of zebrafish larvae parallel to B_0_ field remains unclear, it provides us an intriguing animal model to study the interaction of MF with biological processes.

Though the specific mechanism underlying MF-induced otolith fusion remains to be clarified, we have made two key observations that might shed light on the potential contributing factors. First, when the zebrafish larvae were anesthetized or embedded in agarose, the occurrence of otolith fusion under high MF was significantly reduced, which highlights the importance of the deviation from the magnetic orientation of larvae body under MF exposure to develop the single-otolith phenotype. When the larvae bodies are not aligned to the B_0_ field due to active swimming or passive airflow movement, the magnetic dipole moment intending to line up with the B_0_ field may produce a torque to detach the otoliths from their tethers (the hair cells). The magnetic dipole moment can be varied when the larvae swim across the inhomogeneous MF or by the fast-switch gradients of the pseudo-MR sequence. However, the lack of otolith fusion in the anesthetized larvae under pseudo-MR sequence indicated that the torque created by altered magnetic dipole moment may not be sufficient to cause otolith fusion. On the other hand, exposure of chorionated embryos embedded in agarose that were not anesthetized resulted in fused otoliths. Agarose-embedded embryos still surrounded by the chorion are able to conduct embryonic movements inside the chorionic envelope (despite immobile in the larger-scale arena), which could be regarded as a facilitating feature to accomplish the fusion of otoliths under high MF. Stooke-Vaughan *et al*.^22^ reported a minor role of embryonic movement in otolith formation, but the potential interaction between this type of movement and high MF remains unknown. Nevertheless, we reported a greater occurrence of the MF-induced otolith fusion in older embryos and larvae, which do not exhibit this particular motion. Besides allowing for motion, the chorion might provide sufficient oxygen in the chorionic fluid for the moving embryo, while hatched larvae embedded in agarose could be affected more severely by the reduced oxygen supply in the solidified agar medium.

Secondly, anesthetized and agarose-embedded larvae could present lower level of metabolism compared to larvae kept in fresh embryonic medium (oxygen consumption is presumably reduced for larvae kept in the more solid medium agarose, as well as in larvae which are anesthetized and breath at lower rates), which may contribute to the suppression of otolith fusion. Previous studies have shown that mobility of the hair cell kinocilia directly interfered with the formation of the otolith during development^30^. Although the detailed features of cilia mobility in zebrafish otic vesicle remain disputed^22^,^26^,^30^,^48^, it is plausible that fluidic mobility dampens upon reduced oxygen supplies. Suppressed mobility would reduce the movement of otoliths at the microscopic level, which may serve as a crucial momentum for otoliths to be untetherred from hair cells under MF. As observed by Inoue *et al*.^49^, once otoliths are in close proximity they are able to fuse. Additionally, we observed that gentamicin-treated larvae (which caused high level apoptosis in hair cells) exposed to a MF showed a decrease occurrence rate of otolith fusion in comparison to non-treated control larvae group (Fig. 6a). This observation suggests that functional hair cells might be necessary to enable MF-induced otolith fusion when accompanied with the larvae body movement. In order to directly test cilia mobility in the otic vesicles of living zebrafish lavae under a high MF, a specific MRI compatible microscopic imaging method should be developed. Future work could be intended to test high MF impact on zebrafish mutants displaying two otoliths in their otic vesicles, but with malfunctioned kinocilia.

In conclusion, we described how the high MF exposure to zebrafish larvae led to a phenotype of fused otoliths and a subsequent immotile-circling behavior of the larvae. The translation of this phenotype to the human inner ear is nonetheless fairly unlikely. In contrast to the pair of otoliths in the otic vesicle of zebrafish, human inner ears have a multitude of small calcite-based nanocomposites (otoconia) allocated in a well-established otolithic membrane over the maculae^50^ (Fig. 1a). Therefore, given the differences between these two systems, high MF could have a different impact on the vestibular organ of humans, if so. In some clinical cases, benign paroxysmal positional vertigo^18^, a vestibular disorder in humans, is caused by detached otoconia either free floating in the endolymph or attached to the semicircular canal cupula, most commonly of the posterior semicircular canal. The vestibular anatomy that characterizes this syndrome resembles the MF-induced phenotype in zebrafish, but no reports showed that this syndrome can be directly caused by MF exposure in humans. Although human subjects experiencing vertigo or nystagmus during the MRI scanner have been recently reported^16^,^17^, the proposed mechanism does not involve displacement of otoconia, and therefore the direct impact of high MF on the zebrafish otolith might not translate to other vertebrates. Future work to evaluate the direct MF impact on mammals should provide more detailed understanding of the MF interaction with the biological processes at the microscopic level.

## Methods

### Zebrafish lines

Wild type zebrafish embryos and larvae (Tübingen strain from the Tübingen zebrafish stock center) were used in our experiments reported in this study. Other wild type strains (AB and WIK) were also tested to avoid strain specific response bias (data not shown). Zebrafish were maintained and raised as described previously^23^. Fertilized eggs were collected immediately after spawning and were raised in E3 solution at 28 °C.

### Exposure conditions under the magnetic field

Embryos and larvae between 10 hpf and 6 dpf were transferred at the beginning of each experiment, in Petri dishes to the horizontal bore of a 14T magnet (Agilent, with a Bruker Biospin AVIII console system) equipped with a 12 cm gradient set, capable of providing 100 G/cm with a rise time of 150 μs (Resonance Research). Zebrafish larvae were maintained under the influence of the static MF for an exposure time ranging from 2 to 62 hours. Supplementary Fig. 3 provides specific information about all time windows that have been used in the experiments with zebrafish embryo/larvae. A closed loop heating system was used during all the exposures to keep embryos between 27 °C and 29 °C. After exposure, embryos were either transferred to the incubator for future visualization or processed for immunohistochemistry. To test different scenarios inside the bore of the magnet, we studied 3 different environmental conditions: embryos/larvae in a first group were exposed to the MF in dishes containing E3 solution; embryos/larvae in a second group were exposed to the MF embedded in 0.8% low melting agarose; a third group of larvae was exposed to the MF in dishes containing E3 medium together with 0.04% MS-222 (anesthetic). Larvae exposed in agarose or in anesthetic were immediately transferred to a fresh new medium after the exposure. All the exposures were performed under a static 14 T MF (iso-center of the MRI scanner), with the exception of MF dependence experiments, which took place at different distances from the iso-center. The scanner was running no sequences during the exposures, with the exception of one experiment performed to test the influence of MF gradients. In this experiment, gradient coils around the bore of the magnet participated switching on and off additional MF gradients (RF coils were not used). The pseudo 3D fast low angle shot (FLASH) MR sequence was run with the following parameters: TR: 50 ms, TE: 2.5 ms, Matrix: 256 × 270 × 270, FOV: 3 × 3 × 3 cm (30° was added for the FOV alignment from the magnet geometric X, Y, Z axes), total running time was 2 hour 1 min 30 sec. The gradients were set ranging from 12 to 40 G/cm along the X, Y, Z axes and were repeated every TR (50 ms). Except for this specific condition that demanded the participation of additional hardware, the room accommodating the MRI scanner was silent. The room light was switched off after placement of the samples into the bore of the magnet and remained off until the end of the exposure.

### Video recording inside the MR scanner

A basic camera (Conrad RS-OV7949–1818) was adapted in-house to withstand the high MF. Infrared light (SFH4289 LED) was used as light source inside the bore of the magnet. Eight minutes videos were taken every half an hour to assess the motion of larvae under the influence of high MF.

### Gentamicin treatment of zebrafish larvae

To better characterize the MF effect on the otic vesicle of zebrafish, larvae were treated with the ototoxic aminoglycoside gentamicin^51^ to target the main biological structures implicated in otolith tethering (hair cells). 3 dpf larvae were treated with 50 μM, 100 μM and 500 μM gentamicin for 24 h previous to MF exposure. Animals were exposed to the 14 T MF during the last 2 h of treatment. After the exposure, animals were immediately stained with Acridine Orange (AO) for visualization of apoptotic cells. Control larvae were treated for the same time and then processed for staining.

### Staining procedures

#### Acridine Orange live staining

Apoptosis in zebrafish larvae was assessed with the vital dye Acridine Orange (AO)^52^. Larvae were incubated in 2 μg/mL AO for 20 min, quickly washed and anesthetized in MS-222 (1–3 min) and mounted in low melting agarose for confocal imaging. AO is usually reported at some extent in every living sample^53^,^54^,^55^, therefore comparison with control samples becomes necessary to attribute the apoptotic effect to a certain compound. The signal that we observed in the epithelium of the otic vesicles could be the result of non-specific staining or actual apoptosis in the proliferating tissue^56^,^57^.

#### Anti-acetylated tubulin staining

For anatomical visualization of the otic vesicle and well-defined hair cell structure, zebrafish larvae were stained with antibodies targeting anti acetylated tubulin. 4 dpf larvae were anesthetized with MS-222 (0,04%), fixed in 4% PFA and kept at 4 °C overnight. Methanol series (25%–50%–75%–100%) were accomplished in Eppendorf tubes containing the embryos, and samples were stored at −20 °C until the next working day. Permeabilization was performed by cold acetone immersion for 20 min at −20 °C. Methanol washout took place in series of 10 min (75%–50%–25%) plus 5 washouts of 5 min PBSTw (0, 1% Tween20 in PBS). Proteinase K (10 μg/mL in PBSTw) was added to the samples (during 5 min per dpf) at room temperature to break cross linkages and expose the antigen, followed by a quick double wash in PBST. Postfixation was performed with 4% PFA for 20 min and 3 washouts of 5 min each PBSTw followed. At this stage, samples were moved to a 24 well plate, placing 4 to 8 larvae per well. Blocking was accomplished by immersion in 10% NGS (in PBSTw) for 5 hours at room temperature. We used anti-AcTub as primary antibody in a dilution of 1:1000 in 10% NGS, which was kept overnight at 4 °C. Next day, primary antibody was washed out with PBST 6 times for 30 min each. Secondary antibody (anti mouse Cy3, in a dilution of 1:250) was then added and kept in darkness at 4 °C overnight. Next morning, samples were washed with PBST (5 times 10 min each). We included a post fixation step for 20 min in PFA (to prevent antibody/antigen complexes to be washed away) at room temperature, followed by 3 washes in PBSTw for 5 min each. After washed, glycerol series were performed in the samples (25%–50%–75%–80%) and they were stored in 80% glycerol, in the dark, for future mounting in the slides.

### Live imaging

Non-chemically treated larvae were anesthetized in MS-222 and imaged, without mounting, at low magnification (5×, 10×, 20×) using Zeiss 4750, Leica M205FA and Carl Zeiss Axiovert 40cfl microscopes. Embryos/larvae were observed to assess the vestibular phenotype, which is defined as either a bilateral vestibular effect (both ears presenting the fused phenotype), a unilateral vestibular effect (one ear presenting the fused phenotype) or no effect in any of the two ears. Wild type and AO stained larvae were anesthetized in MS-222, embedded in 0.8% low melting agarose on glass bottom dishes and imaged using confocal microscopy (Zeiss LSM 780 NLO) using 10× and 25× water immersion lenses.

### Confocal microscopy of immunostained samples

For confocal imaging, immune-stained larvae were mounted in 80% glycerol on slides and cover slipped. Larvae were imaged with 25× and 40× water immersion objectives using a Zeiss LSM 780 NLO confocal microscope. Zeiss software was used for snaps and stacks acquisition. All images were processed using ImageJ free software. 3D and Z project tools were used for three-dimensional reconstruction of stacks and overlay of images, respectively.

### Behavioral assessment

Position of the body while standing (vertical ventro-dorsal axis vs. vertical lateral axis), amount of time that larvae spent in movement (active vs. inactive) and type of movement described (mostly in straight lines vs. mostly in circles) were considered. For assessment of the swimming patterns, 3–5 dpf larvae were allowed to swim freely in dishes and were tracked using DanioVisionTM (Noldus). As a routine, 2 min videos were performed for evaluation of the balance and movement of each experimental group one day after the exposure. In case of fish that were exposed to the MF while anesthetized, the anesthetic was washed out immediately after exposure by changing the larvae to a fresh new medium. Zebrafish larvae got completely awake 5 minutes after the washout of the anesthetic.

### Statistical analysis

All statistics were performed using a two-tail T-Test, entering the number of trials as the number of samples, and the percentage of larvae with fused otoliths in each trial as the value for each sample. Error is shown in the graphs as the standard error.

## Additional Information

**How to cite this article**: Roldán, P. P. *et al*. High magnetic field induced otolith fusion in the zebrafish larvae. *Sci. Rep.* **6**, 24151; doi: 10.1038/srep24151 (2016).

## Supplementary Material

Supplementary Information

Supplementary video 1

Supplementary video 2

Supplementary video 3

Supplementary video 4

Supplementary video 5

Supplementary video 6

Supplementary video 7

Supplementary video 8

Supplementary video 9
