# Supplementary material for: High magnetic field induced otolith fusion in the zebrafish larvae
Source: Sci Rep. 2016 Apr 11;6:24151. doi: 10.1038/srep24151 (PMC4827070; doi:10.1038/srep24151)
Supplement: Supplementary Information [file srep24151-s1.pdf]

Supplementary Figures and Video Legends

High magnetic field induced otolith fusion in the zebrafish larvae

Patricia Pais Roldán[1,2], Ajeet Pratap Singh[3], Hildegard Schulz[1], Xin Yu[1*]

[1] Max Planck Institute for Biological Cybernetics; [2] IMPRS for Cognitive and Systems Neuroscience; [3] Max Planck Institute for Developmental Biology, Tübingen, 72076, Germany

Running title: Zebrafish larvae otolith fusion under 14T MRI scanner

* Corresponding author

Email: xin.yu@tuebingen.mpg.de

Keywords:

Otolith, Magnetic Field, Magnetic Resonance Imaging, zebrafish larvae

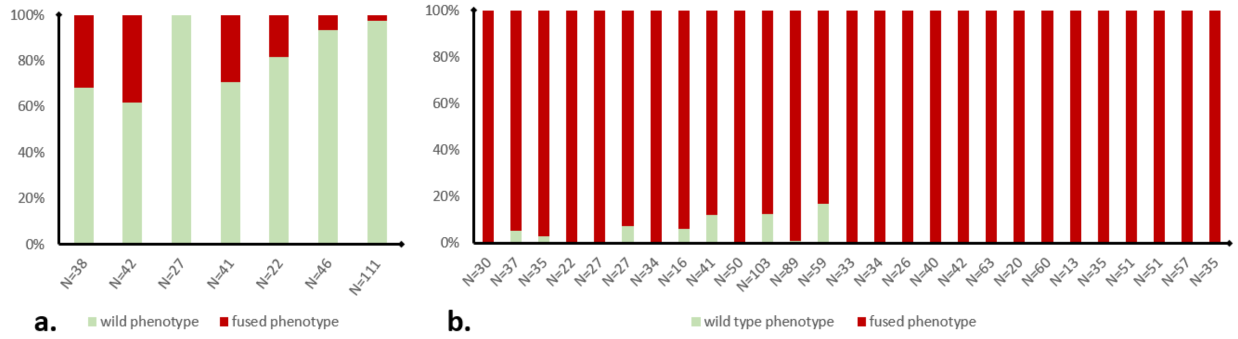

Supplementary Fig. 1. Repeatability of the MF-induced fusion of otoliths in zebrafish larvae. a. MF response (fusion of otoliths) in larvae younger than 24 hpf. **b**. MF response in larvae older than 24 hpf. A significant difference in the response of both groups can be observed (more susceptibility in larvae older than 24 hpf). Vertical axes represent the percentage of larvae with fused otoliths (red color) and larvae with normal separate otoliths (green color) upon exposure to the static 14T MF. Each column represents one trial or experiment. The number of samples (N) included in each one of the trials is shown on the X axis.

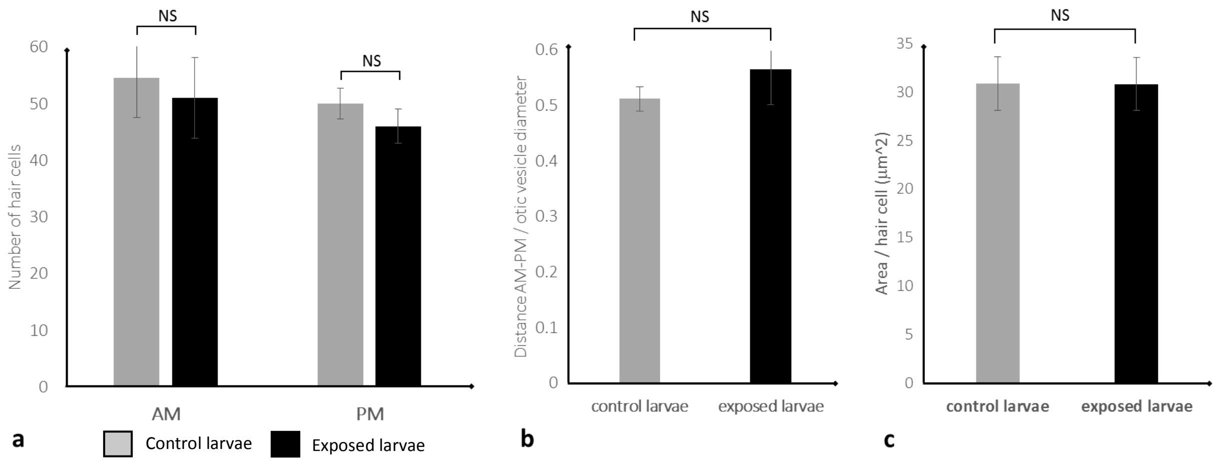

Supplementary Fig. 2. Characterization of the otic vesicle in control and exposed larvae. Figure **a**. shows the number of hair cells in the anterior macula (AM) and posterior macula (PM) in control and exposed larvae. Figure **b.** represents the relative distance between the two maculae (average = 93 and 96 μm in control and exposed respectively) with respect to the diameter of the otic vesicle (average diameter= 182 and 173 μm in control and exposed animals respectively). Figure **c.** shows the approximate area in the otic vesicle that is occupied by each hair cell in the anterior macula in control and exposed larvae (average anterior macula area = 1575 and 1680 μm^2 in control and exposed larvae, respectively). No significant differences between the 2 groups were found in either the number of hair cells, the distance between maculae or in how spread the hair cells in the macula are. Larvae for this experiment were fixated at 4 dpf and stained with anti-acetylated tubulin. All images were acquired with confocal microscopy and processed with ImageJ. The number of animals studied at cellular level (n) was 8 for graph **a** and 5 for graphs **b** and **c**.

(dpf)	0	0	0	0	0	1	1	1	2	2	2	2	3	3	3	3	3	4	4	4	5	5	6	6
(hpf)	8	10	11	12	20	24	30	36	48	51	52	57	72	74	75	78	86	96	98	99	120	123	144	146
	x	x	x	x	x	x	x	x	x	x	x													
	x	x	x	x	x	x																		
		x	x	x	x	x	x	x	x															
		x	x	x	x	x																		
			x	x	x	x	x	x	x	x	x	x												
				x	x	x	x	x	x															
				x	x	x	x	x																
				x	x	x																		
					x	x	x	x	x															
					x	x																		
						x	x	x	x															
						x	x	x																
						x	x																	
								x	x															
									x	x	x	x	x											
									x	x														
													x	x	x	x	x							
													x	x	x	x								
													x	x	x									
													x	x										
																		x	x	x				
																		x	x					
																					x	x		
																							x	x
(dpf)	0	0	0	0	0	1	1	1	2	2	2	2	3	3	3	3	3	4	4	4	5	5	6	6
(hpf)	8	10	11	12	20	24	30	36	48	51	52	57	72	74	75	78	86	96	98	99	120	123	144	146

Supplementary Fig. 3. Times of exposure to the high MF. The table shows the different possibilities of exposure time that have been used along the studies. The top and bottom number guides show the age of zebrafish larvae, and “x” inside the boxes show the limited period of life in which the embryos/larvae have been exposed. dpf = days post fertilization; hpf = hours post fertilization.

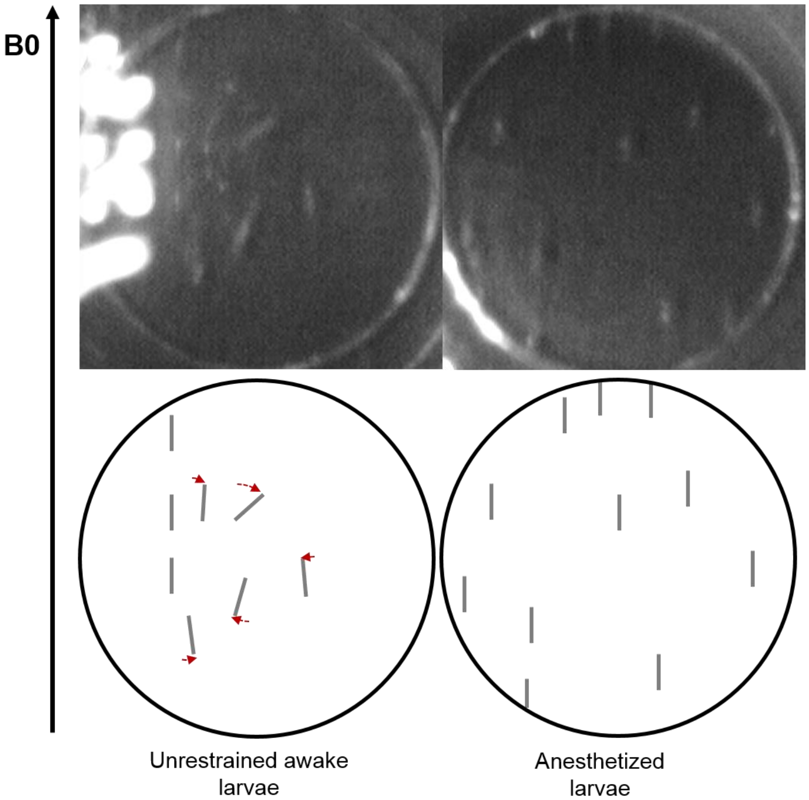

Supplementary Fig. 4. Body orientation of the zebrafish larvae inside the bore of the 14T magnet. The upper images show a snapshot of the larvae under high MF (taken from videos performed inside the scanner) and a schematic simplifying the position of their bodies is presented below. Awake larvae change the orientation of their body with respect to B0 when they move (red arrows show the deviation of the body of moving larvae from the B0 direction). In contrast, anesthetized larvae remain immobile and exhibit alignment with the MF during all the exposure time.

VIDEOS:

Supplementary video 1. Swim of wild type zebrafish larvae. Normal larvae swam in frequent short straight patterns.

Supplementary video 2. Swim of exposed zebrafish larvae. After exposure, most larvae failed to balance correctly while standing (lying sideways) and those who swam had a fast circling pattern (readily observable at second 18).

Supplementary video 3. Behavior of awake zebrafish larvae inside the MRI scanner. Awake larvae swim normally inside the scanner but return to a position with their body oriented in parallel to the MF at resting state.

Supplementary video 4. Behavior of anesthetized zebrafish larvae inside the MRI scanner. Anesthetized larvae aligned their body with the strong MF when they approximate to the iso-center of the scanner (first five seconds of the video) and remain in this orientation for the whole exposure time.

Supplementary Video. 5. Behavior of anesthetized zebrafish larvae inside the MRI when a mild airflow is applied. A mild flow is able to move the anesthetized larvae but only in a translational manner, without modifying the alignment of their body with the high MF.

Supplementary Video. 6. Behavior of anesthetized zebrafish larvae inside the MRI when a strong airflow is applied. A strong flow is able to move the anesthetized larvae disrupting the alignment of their body with the high MF.

Supplementary Video. 7. The 3D reconstruction of the otic vesicle of a control larva. Confocal images were acquired from the otic vesicle stained with anti-acetylated tubulin from a 4 dpf zebrafish larva. Both anterior and posterior maculae can be identified (white arrows) with a set of well-defined hair cells (with darker nuclei and long kinocilia, at the base of the otic vesicle) from the 3D rendering view.

Supplementary Video. 8. The 3D reconstruction of the otic vesicle of an exposed larva. Confocal images were acquired from the otic vesicle stained with anti-acetylated tubulin from a 4 dpf zebrafish larva that was exposed to the MF from 24 to 48 hpf. Both anterior and posterior maculae can be identified (white arrows) with a set of well-defined hair cells (with darker nuclei and long kinocilia, at the base of the otic vesicle) from the 3D rendering view.

Supplementary Video. 9. Behavior of zebrafish larvae treated with 500μM gentamicin inside the MRI scanner. Gentamicin-treated larvae behave similarly to non-treated larvae.
