# Supplementary figures and images for: High magnetic field induced otolith fusion in the zebrafish larvae
Source: Sci Rep. 2016 Apr 11;6:24151. doi: 10.1038/srep24151 (PMC4827070; doi:10.1038/srep24151)

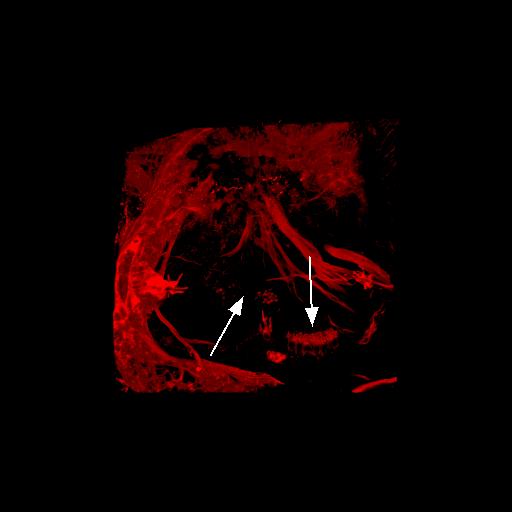

Supplement: Supplementary video 7 [file srep24151-s8.gif]

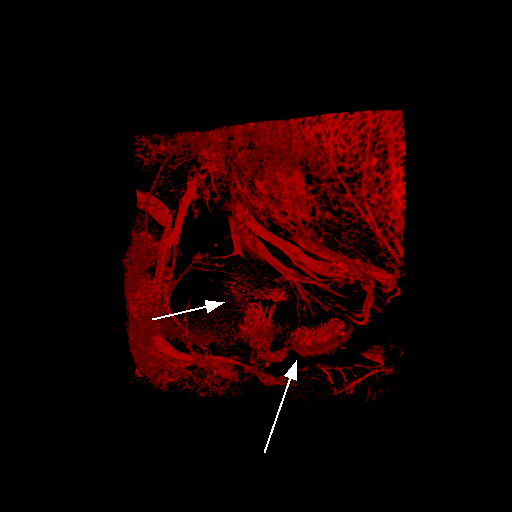

Supplement: Supplementary video 8 [file srep24151-s9.gif]
